# Rural–Urban Inequities in Tuberculosis-Related Practices in Equatorial Guinea

**DOI:** 10.1007/s44197-023-00162-9

**Published:** 2023-10-23

**Authors:** Alba Ayala, Policarpo Ncogo, Juan Eyene, Belén García, Agustín Benito, María Romay-Barja

**Affiliations:** 1 Centro Nacional de Medicina Tropical, Instituto de Salud Carlos III, Madrid, Spain; 2 Fundación Estatal, Salud, Infancia y Bienestar Social (FCSAI), Madrid, Spain; 3 Ministerio de Salud y Bienestar Social, Malabo, Equatorial Guinea; 4 Centro de Investigación Biomédica en Red de Enfermedades Infecciosas (CIBERINFEC), Madrid, Spain

**Keywords:** Tuberculosis, Practices, Health-seeking behaviour, Treatment abandon

## Abstract

**Background:**

Tuberculosis (TB) is one of the leading causes of mortality from a single infectious disease agent. Equatorial Guinea is a country with high estimated TB incidence in 2021 (275 cases per 100,000 population) and low TB case detection (42%). Early diagnosis and prompt treatment are crucial for TB control. Failure to seek adequate health care increases the disease’s transmission and leads to poor treatment outcome, the mortality, even for easily manageable conditions. Information regarding community management of TB and treatment-seeking patterns in Equatorial Guinea is rare. The aim of this study was to explore differences in TB health-seeking behaviour among urban and rural population TB cases in Equatorial Guinea and the factors associated with this behaviour.

**Methods:**

A national cross-sectional study of 770 household caregivers was conducted in 2020 in Equatorial Guinea using multistage stratified sampling. The 284 caregivers that reported having had a TB case in their family were included in this study. A practice index was created. Poisson regression with robust variance was performed with the practices index as dependent variable to assess the factors associated with the health-seeking behaviour.

**Results:**

Most of the cases (65%) have had good TB health-seeking practices. However, 23.2% of TB cases reported having abandoned treatment before 6 months. A higher probability of having good TB practices was observed with being women, aged and living in rural area. Those who were TB cases themselves have heard about TB on the radio, and had high knowledge about TB, hand also good practices.

**Conclusions:**

Disparities in tuberculosis health-seeking behaviour between rural and urban populations highlight the challenges existing in the fight against this infectious disease. The National Tuberculosis Control Program has to reinforce the health system needs to strengthen the follow-up of TB patients taking into account the population at risk of inappropriate TB behaviour.

**Trial Registration:**

Not applicable.

## Introduction

Tuberculosis (TB) is one of the leading causes of mortality from a single infectious disease agent [1]. The World Health Organization (WHO) estimated 9.9 million people infected with TB in 2021 and 13% of mortality rate worldwide [2]. Although there has been a significant reduction in TB-related mortality over the last 3 decades due to improvements in antimicrobials and public health measures, the COVID-19 pandemic has reversed years of progress in providing essential TB services worldwide, causing a large global decline in the number of people newly diagnosed with TB [2].

The microorganism *Mycobacterium tuberculosis* (MTB) that causes TB is an airborne infectious agent that mainly affects the lungs, making pulmonary disease the most common presentation. Despite its severity, tuberculosis is a preventable and curable disease, through early diagnosis, effective treatment with a 6-month drug regimen, and adequate patient support. However, the long length of treatment makes adherence difficult. The global treatment coverage is still low, with great concern about multidrug-resistant TB [2].

Tuberculosis remains one of the major health problems in Equatorial Guinea with a high estimated TB incidence (275 cases per 100,000 inhabitants) in 2021, an estimate mortality rate of 51/100,000, a very low TB case detection (42%) and a high rate of rifampicin resistance in new cases (54%) [3]. However, the social factors associated with these poor outcomes are mainly unknown.

The poor patient adherence in tuberculosis (TB) treatment is considered to be one of the most serious challenges. The National Tuberculosis Control Program (PNLT) is trying to improve TB diagnosis access and treatment adherence by reinforcing free quality diagnostic and treatment services throughout the country. However, TB has biomedical and socio-economic dimensions that are key determinants of TB distribution [4]. Behavioural insights about the population TB health-seeking diagnosis practices and treatment adherence are essential to design targeted interventions to improve access to TB services in Equatorial Guinea. Early diagnosis and prompt treatment are crucial for TB control. Failure to seek adequate health care increases the disease’s transmission and mortality, even for easily manageable conditions [5]. Equatorial Guineans lack important knowledge about TB disease [6]. However, health-seeking behaviour is not only influenced by the patient’s knowledge, social determinants also drive when and where a patient will seek care for TB symptoms [7]. Factors, such as gender, age, economic status, and level of education, have been found to determine health care-seeking behaviour among presumptive TB patients [8, 9]. Furthermore, economic growth and urbanization during the last decades have led to wider gaps between urban and rural areas in Africa. The gap generated by the living area needs to be acknowledged and recognized. It is necessary to have a better understanding of the urban and rural communities’ TB health-seeking practices to implement better disease prevention and control [10, 11].

In Equatorial Guinea, little is known about social determinants of population TB management, treatment-seeking patterns, and adherence. By filling this gap of knowledge, targeted interventions can be introduced to improve treatment outcomes. The aim of this study was to explore differences in TB practices among urban and rural population in Equatorial Guinea and the factors associated with the TB cases behaviour. These insights will support the PNLT in designing contextualized strategies addressed to improve TB prevention and control.

## Methods

### Study Area

Equatorial Guinea has an area of 28,051.5 km2 and a population of 1,225,377 inhabitants [12], with 47.6% women and 23.9% rural population. The number of households is 262.157 with an average size of 4.3 people [12]. Equatorial Guinea presents two regions: a Mainland Region, which borders with Cameroon to the north and Gabon to the south and east; and an Insular Region consisting of two islands, Bioko and Annobon. Bioko is the largest island and is where the country's capital, Malabo, is located. The Mainland Region is divided into four provinces: Centro Sur, Kie-Ntem, Wele-Nzas, and Litoral.

### Study Design and Sampling

Following the validated WHO guide [13], a questionnaire on Knowledge, Attitudes and Practices (KAP) was designed to determine tuberculosis knowledge, attitudes, and practices in Equatorial Guinea rural and urban households. Using this questionnaire, data were collected in October 2020 in 55 communities of the mainland region and Bioko Island. A multistage cluster random sampling was implemented stratified by area of residence (rural/urban). Methodological aspects have been previously described [6]. A sample size of 770 caregivers was calculated using the following formula: $n\, = \,\left[ {{\text{DEFF}} \times {\text{Np}}\left( {{1} - {\text{p}}} \right)} \right]/ \, \left[ {({\text{d2}}/{\text{Z21}} - \alpha /{2} \times \left( {{\text{N}} - {1}} \right)\, + \,{\text{p}} \times \left( {{1} - {\text{p}}} \right)} \right]$, where N was the latest data published from the Equatorial Guinea population census [12], considering a proportion (p) of good knowledge about TB of 50%, a confidence level of 95%, a precision of 5% (d2), and a correction factor for the design effect for complex samples of two (DEFF). The inclusion criteria were people with 18 years and older. For this study, those caregivers that reported having had a TB case in their family were selected (*n* = 284).

### Variables

Sociodemographic characteristics of TB cases and characteristics of the household conditions were collected. Variables about type of roof and floor material, source of water, electricity assess, sanitation facilities, and having TV or radio were used to calculate a wealth index through principal component analysis [14, 15].

Knowledge index was performed through 13 correct questions about symptoms, risk perception, transmission mechanisms, prevention, and treatment of the TB disease [6]. The knowledge score was calculated by adding values of the correct questions, with greater values (ranged from 0 to 13) indicating good knowledge. The median values of the knowledge index (cut-off = 8) were used to classify it into low or high categories.

A practice index was built taking into account questions about where people go for the first symptoms of TB, intake of treatment and adherence. The following correct answers were coded as 1: going to a health facility (hospital or health center) at the first TB symptoms, taking TB treatment, having taken the treatment during 6 or more months; and the incorrect answers were coded as zero. The answers were summed, resulting in an index with a range of 0 to 4. This index was divided into bad and good practices according to the median (2.6).

### Data Analysis

A descriptive analysis was conducted to describe sociodemographic characteristics of TB cases, their health-seeking behaviour, information sources, and their practices related with TB, using absolute and relative frequencies by area (rural and urban). Chi-square tests were calculated to analyse the differences between areas.

Poisson regressions with robust variance were performed to calculate prevalence ratios (PR) and 95% confidence intervals (95% CI), to avoid odds ratio overestimations for prevalence rates above 10% [16]. Forward stepwise procedure was applied for the model selection. The dependent variable was the dichotomous practice index and the independent variables were the variables included in the sociodemographic characteristics, usual health-seeking behaviour, and information sources.

## Results

### Sociodemographic Characteristics by Area

Out of the 770 caregivers interviewed, 284 (37%) reported having had a TB case in the family. Most of the TB reported cases lived in an urban area (78%), were women (49.7%) with a mean age of 43.1 (standard deviation = 17.1). TB cases in rural areas were poorer (59.1%) than in urban areas (6.4%, *p* < 0.001), while house overcrowding was found greater in the urban area (61.9%) than in the rural area (40.9%, *p* = 0.003). There were no significant differences in TB knowledge by area (Table 1).

**Table 1:** Sociodemographic characteristics of TB cases by area

	Rural	Urban	*p* value
	*n* = 66 (%)	*n* = 218 (%)	
*Sex*			0.290
Man	37 (56.1)	106 (48.6)	
Woman	29 (43.9)	112 (51.4)	
*Age*			0.505
< 38 years	21 (31.8)	83 (38.1)	
38–53 years	24 (36.4)	80 (36.7)	
> = 54 years	21 (31.8)	55 (25.2)	
*Marital status*			0.591
Not married	29 (43.9)	104 (47.7)	
Married	37 (56.1)	114 (52.3)	
*Ethnicity*			0.055
Fang	58 (87.9)	179 (82.1)	
Other	8 (12.1)	39 (17.9)	
*Religion*			0.269
Catholic	59 (89.4)	172 (78.9)	
Other	7 (10.6)	46 (21.1)	
*Educational status*			0.075
Primary and lower	26 (39.4)	66 (30.3)	
Secondary	19 (28.8)	48 (22.0)	
Above secondary	21 (31.8)	104 (47.7)	
*Current working*			0.310
Yes	16 (24.2)	67 (30.7)	
No	50 (75.8)	151 (69.3)	
*Wealth*			< 0.001
Poorest	39 (59.1)	14 (6.4)	
Second	17 (25.8)	34 (15.6)	
Middle	3 (4.5)	54 (24.8)	
Fourth	6 (9.1)	55 (25.2)	
Richest	1 (1.5)	61 (28.0)	
*Overcrowding index*			0.003
< = 1.4 points	39 (59.1)	83 (38.1)	
> 1.4 points	27 (40.9)	135 (61.9)	
*Knowledge score*			0.995
Low	36 (54.5)	119 (54.6)	
High	30 (45.5)	99 (45.4)	

### Health-Seeking Behaviour and Information Sources by Area

Most respondents (83.8%) said that they used to go the hospital to treat their health problems, without significant differences by area (Table 2). When asked about the time and transport normally used to reach a health facility, TB cases in rural areas reported taking longer to reach a health facility than those in urban areas (*p* < 0.001). Most respondents reported going to health facilities by taxi, while walking was less frequent in rural than in urban areas (*p* < 0.001). Respondents reported hearing about TB mainly from family and health workers, and television was significantly more frequent source of information in urban area (30.7%, *p* = 0.003).

**Table 2:** Usual care-seeking behaviour and information sources

	Rural	Urban	*p* value
	*n* (%)	*n* (%)	
*Where do you usually go if you have a health problem?*			
Hospital	57 (86.4)	181 (83.0)	0.519
Health center	10 (15.2)	23 (10.6)	0.307
Private clinic	8 (12.1)	29 (13.3)	0.803
Traditional healer	5 (7.6)	14 (6.4)	0.742
Pharmacy	0 (0.0)	7 (3.2)	0.140
Other	1 (1.5)	4 (1.8)	0.863
*How do you normally go to the Health Center or hospital?*			< 0.001
Taxi	37 (56.1)	130 (59.6)	
Car	16 (24.2)	44 (20.2)	
Walking	6 (9.1)	44 (20.2)	
Motorcycle	1 (1.5)	0 (0.0)	
Do not know	6 (9.1)	0 (0.0)	
*How long does it take to get to your nearest Health Center or hospital?*			< 0.001
< 1 h	32 (48.5)	176 (80.7)	
1 h or more	34 (51.5)	42 (19.3)	
*How many times a year do you seek medical care at the Health Center or hospital?*			0.182
Once a year or more	49 (74.2)	124 (56.9)	
Less than once a year	17 (25.8)	180 (82.6)	
*Where have you heard about TB?*			
Family and friends	43 (65.2)	121 (55.5)	0.165
Health workers	41 (62.1)	118 (54.1)	0.252
Radio	11 (16.7)	41 (18.8)	0.694
TV	8 (12.1)	67 (30.7)	0.003
Other	8 (12.1)	24 (11.0)	0.069

### Health-Seeking Behaviour by Steps

**Fig. 1:**
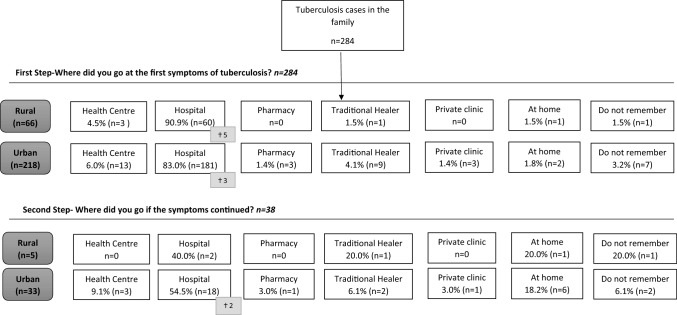
Tuberculosis health-seeking behaviour by area in Equatorial Guinea

Regarding the health-seeking behaviour of TB cases at the first step, most respondents said that they went to the hospital at the first symptoms (85.2%), with significant differences by area (rural 90.9% and urban 83.0%, *p* = 0.041). The health center (5.6%) was the second most-mentioned option and 3.5% of the TB cases said that they went to a traditional healer, with no differences by area. TB cases whose symptoms continued after their first visit (13.4%) mentioned having going to hospital as a second step in seeking TB care (52.6%), an option significantly lower in rural than in urban area (*p* = 0.046), while 18.4% decided to stay at home, and visiting a traditional healer increased as an option compared to the first step (7.9%) (Fig. 1).

### Tuberculosis Treatment Adherence

**Fig. 2:**
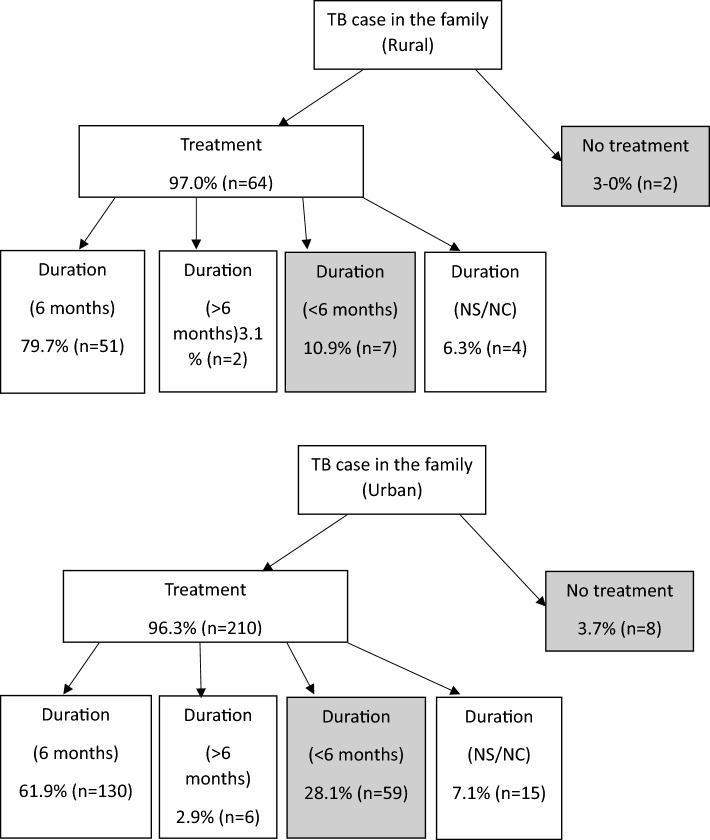
Tuberculosis treatment adherence reported by area

According with the interviewed, most TB cases (96.5%) received treatment. Anti-tuberculosis treatment was the most frequent mentioned (80.6%) and 12.7% said that they did not know the treatment taken. While most cases reported having been on treatment for 6 months (80% of cases in rural area and 62% in urban), 23.2% of TB cases reported having abandoned treatment before (Fig. 2). TB treatment abandonment was significantly lower in rural areas (11%) than in urban ones (28%) *p* = 0.004. The most frequently mentioned reason for treatment abandonment was that they no longer had symptoms (28.2%). Some cases, 3.5% of TB cases, reported not having taken any treatment.

### TB Health-Seeking Practice Index and Associated Factors

Most of the cases (65%) reported have had good TB health practices, with most of the respondents correctly answered at least three of the four questions included in the index. Good TB health practices were significantly more frequent in respondents from rural (79%) than urban area (60% *p* = 0.005).

Regarding the factors associated with good practices of TB reported cases (Table 3), a higher probability of having good practices was observed in females, the aged and those who were TB cases themselves (PR: 1.560), those who have heard about TB on the radio (PR: 1.265, 95%CI 1.033–1.548), those living in rural areas (PR: 1.271), and having high knowledge about TB (PR: 1.064). Bad health TB practices were associated with those who have heard about TB on television (PR: 0.683, 95%CI 0.532–0.878) and usually went to a private clinic when they have a health problem (PR: 0.711, 95%CI 0.509–0.994).

**Table 3:** Factors associated with TB health-seeking practices in Equatorial Guinea

Variable (reference)	Unadjusted model			Adjusted model		
	PR	CI95%		aPR	CI95%	
Age	1.002	0.997	1.007	1.000	0.995	1.005
*Sex (Male)*						
Female	1.095	0.921	1.302	1.072	0.909	1.265
TB cases themselves (TB in the family)	1.529	1.333	1.755	1.560	1.326	1.836
*Where have you heard about TB?- (No)*						
Television	0.730	0.574	0.930	0.683	0.532	0.878
Radio	1.131	0.926	1.380	1.265	1.033	1.548
*Go to private clinic for a health problem (No)*						
Yes	0.684	0.477	0.981	0.711	0.509	0.994
*Area (Urban)*						
Rural	1.311	1.111	1.547	1.271	1.078	1.497
*Knowledge (Low)*						
High	1.089	0.916	1.294	1.064	0.902	1.255

## Discussion

This study highlights relevant behavioural insights about TB cases treatment-seeking behaviour and adherence by area in Equatorial Guinea. Rural TB cases were poorer, had lower educational level, and took longer to reach a health facility than urban cases. However, despite these inequities, they showed a better TB practice index and higher treatment adherence. Age, sex, source of information, and TB knowledge were other factors found associated with good TB care practices.

The high percentage of TB cases who attended a health facility was similar in Equatorial Guinea than in other countries [17–20], and “not knowing where to go” was the most frequent reason for seeking alternative TB care in Equatorial Guinea and other countries of the region [21, 22].

Despite the adequate TB care behaviour observed at the first symptoms in both areas, hospital utilization was higher in rural than in urban areas at the onset of TB symptoms in Equatorial Guinea. Significant differences by area in approaching a health care facility first have been found in other studies [10, 23, 24] where rural TB cases also used government facilities more frequent than urban cases, being the lack of other alternatives in rural areas together with considering the hospital as the best place for treatment, the most frequent reasons for this behaviour. However, the percentage of cases who decided to manage the disease elsewhere increases considerably after the first consultation, with a worrying increase in those who decide to visit a traditional healer or staying at home. Second-step change of health care provider has been also found due to the dissatisfaction with the first health care service [23], while the most common reason for staying at home without taking any action was that having a cough for more than 2 weeks was not considered a serious symptom [7]. This behaviour negatively affects the early detection of cases and the rapid starting of appropriate treatment, leading to a delay in patient improvement and disease control.

The high proportion of TB treatment abandonment reported in this study (23%) is similar to that found in a previous study (21%) of patients attending TB referral units in Equatorial Guinea [25]. One of the most frequently mentioned reasons for treatment abandonment was the end of symptoms. In a country where multidrug resistance in new cases was three times higher than in the rest of Africa [26] improving the knowledge and relevance of treatment duration should be a priority. However, treatment abandon is lower in rural than in urban areas. After the first month of hospitalization, patients in rural areas have to go to the hospital once a month to get the rest of their treatment, while those in urban areas have to go every week. This could explain why adherence to TB treatment is significantly higher in rural than urban areas in Equatorial Guinea, as patients in urban areas may be demotivated by having to go to hospital so often to get their treatment during the following five months. Although an active treatment abandonment tracing program has been put in place, Directly Observed Therapy (DOT) is not implemented in Equatorial Guinea and TB patients must continue treatment at home on their own where they find no community support, no education programs and no good counselling.

Other factors associated with having good TB care practices were being female and age. Being older was also associated with better health TB behaviour and less delay in searching adequate treatment in Uganda [27]. While women use to frequent health services more in Ethiopia [25], other studies found that women used to seek care for TB when symptoms become unbearable [28–31]. Despite the low TB knowledge found among Equatorial Guinean [6], to have a better knowledge about TB transmission, diagnostic and treatment were also associated with a better TB health-seeking behaviour in Equatorial Guinea, as in the other studies [32, 33].

This study shows that cases who have heard about TB on the television have bad health-seeking practices. Hearing about TB on the television have been also found associated with having high stigma in Equatorial Guinea [6]. However, receiving information about TB over the radio was associated with a good TB health-seeking behaviour, as messages on the radio seem to be better adapted to the context and the local audiences [33].

Furthermore, frequently, a private clinic was associated with bad TB index behaviour. Some studies have found that many private practitioners are poorly trained in the diagnosis and treatment of TB and lack the communication skills required to motivate the patient for treatment intake and adherence [11].

This study has some limitations. First, it is a cross-sectional study, so the findings may not be generally applicable to different contexts. Second, there could be a problem of recall when the TB onset occurred some time ago, especially when caregivers reported the case of a family member.

## Conclusions

The disparities in tuberculosis health-seeking behaviour between rural and urban populations highlight the challenges existing in the fight against this infectious disease. Understanding the multifaceted nature of these differences is essential for developing effective interventions and strategies that would address the needs of each setting. Rural cases have better TB health-seeking behaviours and greater adherence to treatment. However, treatment abandonment is high in both areas and interventions need to take into account the determinants of the population at risk of having inappropriate TB behaviour like being urban, men, being young and have low TB knowledge. The health system needs to be strengthened in the follow-up of TB patients but only through collaborative and context-specific strategies would it be possible to reduce incidence, and improve adherence and treatment outcomes.

## Data Availability

The authors confirm that data supporting the findings of this study are available within the article.

## Abbreviations

TB: Tuberculosis
PNLT: The National Tuberculosis Control Program
TV: Television
DOT: Directly Observed Therapy
